# Protocol optimization and physiological specificity of ASL-measured reactive hyperemia in skeletal muscle

**DOI:** 10.1186/1532-429X-16-S1-P37

**Published:** 2014-01-16

**Authors:** Hou-Jen Chen, Graham A Wright

**Affiliations:** 1 Imaging Research, Sunnybrook Research Institute, Toronto, Ontario, Canada; 2 Medical Biophysics, University of Toronto, Toronto, Ontario, Canada

## Background

Evidence suggests that microvascular function may be compromised in Peripheral Arterial Disease (PAD) due to associated endothelial dysfunction. While revascularization is recommended for PAD patients with macrovascular occlusions, a solution for endothelial dysfunction remains unavailable. For a possible endothelium-targeted therapy to be applied, the burden of endothelial dysfunction at different disease stages needs to be characterized. Microvascular function is commonly tested through measurement of reactive hyperemia induced by a short period of ischemia. However, patients with endothelial dysfunction may still demonstrate hyperemia due to build-up of metabolites causing relaxation on their relatively intact vascular smooth muscles. We measured and characterized the reactive hyperemia after various durations of arterial occlusion in the leg, with the goal to reduce the influence of tissue metabolites on detection of endothelial dysfunction.

## Methods

Following a protocol approved by Sunnybrook's Research Ethics Board, seven healthy subjects each underwent 2, 3, and 5 minutes of ischemia. Five of them also participated a1-minute ischemia experiment. Ischemia was achieved by inflation to 220 mmHg of an air-cuff on the thigh. ASL of the calf was performed on a 3T scanner with an 8-channel cardiac array. The ASL scan started 30 seconds before deflation of the air-cuff and continued for 2 minutes. The ASL used here was FAIR-Q2TIPS with 1400 ms of post-labeling delay. Perfusion responses were quantified in the soleus muscle.

## Results

The tabulated data are consistent with earlier findings indicating that the early and late phases of reactive hyperemia may be mediated by different mechanisms. Our data show that the peak responses did not differ significantly for occlusions greater than 1 min, supporting a recent hypothesis that endothelium-dependent vasodilation, mediated by fast depletion of oxygen, may dominate in the early phase. On the other hand, accumulation of tissue metabolites is known to prolong the hyperemic response after longer occlusions, which is also consistent with our results. In addition to the endothelial specificity of the stress protocol, an optimal protocol should also induce responses that are suitably captured by ASL. Because the ASL signal is proportional to perfusion, a large hyperemia is preferred. Moreover, a response that lasts for around 10 seconds would suffice to avoid the case of missed peak response, given that the sampling rate of ASL is 5 to 10 seconds.

## Conclusions

Reactive hyperemia created by 2 to 3 minutes of arterial occlusion may be a more specific and efficient protocol for endothelial dysfunction.

## Funding

Queen Elizabeth II/Sunnybrook Scholarship in Science and Technology.

**Table 1:** 

Occlusion	Peak Perfusion (ml/100 g/min)	Time-to-Peak (s)	Duration above 60% (s)
1 minute (n = 5)	49.2 ± 10.0	7.7 ± 2.2	6.0 ± 3.2

2 minutes (n = 7)	57.3 ± 10.4	10.8 ± 6.3	14.2 ± 5.1 †

3 minutes (n = 7)	60.8 ± 9.7	19.1 ± 10.0 †	18.6 ± 4.4 †

5 minutes (n = 7)	62.2 ± 6.5 †	22.2 ± 12.1 † ‡	26.6 ± 14.9 †

Values are mean ± SD. † p < 0.05 versus 1-minute occlusion. ‡ p < 0.05 versus 2-minute occlusion.

